# Examining the association between AI-enhanced education and medical students’ self-directed learning using an integrated TAM-UTAUT2 model

**DOI:** 10.3389/fmed.2026.1817255

**Published:** 2026-04-24

**Authors:** Jin Zhu, Chongyuan Guan, Hao Zhang, Lijia Wang, Yuanyuan Zhang, Xiaofei Bian

**Affiliations:** 1 School of Public Health, Dalian Medical University, Dalian, China; 2 Dalian Rehabilitation and Recuperation Center of Joint Logistic Support Force of PLA, Dalian, Liaoning, China; 3 Department of Pediatrics, Dalian Medical University, Dalian, China

**Keywords:** Artificial Intelligence-Assisted, Medical Students, Self-Directed Learning, TAM, UTAUT2

## Abstract

**Background:**

Artificial intelligence (AI)-assisted education has become an increasingly important instructional model in medical education and raises questions about how it shapes students’ self-directed learning processes and technology adoption.

**Objectives and methods:**

This study examined factors associated with self-directed learning and AI-related behavioral intention among medical students using a cross-sectional survey design. A model integrating self-directed learning dimensions with TAM- and UTAUT2-related constructs was tested with 600 valid questionnaires, and data were analyzed using partial least squares structural equation modeling (PLS-SEM) in SmartPLS 4.

**Results:**

The results supported 21 of the 24 hypothesized paths. Motivation emerged as the strongest predictor in the model and was significantly associated with attitude and all three self-directed learning dimensions. Attitude was also significantly associated with self-planning, self-management, and self-monitoring. Self-planning was positively associated with self-management, and self-management was positively associated with self-monitoring. In the technology acceptance pathway, perceived ease of use and perceived usefulness were associated with behavioral intention, and behavioral intention was associated with actual behavior. Facilitating conditions and social influence were also associated with behavioral intention. The model explained substantial variance across key constructs, ranging from 47.6% in actual behavior to 69.9% in self-management.

**Conclusion:**

These findings suggest that motivational support, structured self-directed planning activities, and adequate digital infrastructure may be relevant considerations for AI integration in health sciences education. The study provides preliminary evidence that a model integrating self-directed learning dimensions with TAM and UTAUT2 related constructs may help explain AI-assisted learning behavior in this population and highlights the need for longitudinal research to clarify the directionality of these associations.

## Introduction

Artificial intelligence, a key 21st-century technology, is transforming various fields, including business, science, and healthcare (1), and plays a crucial role in education (2). AI applications, such as ChatGPT and AI-assisted diagnostic tools, are increasingly used in medical education and clinical training across specialties like ophthalmology (3), radiology (4), and residency programs (5). AI offers solutions to educational challenges (6, 7); for example, Fang et al. (5) demonstrated successful use of an AI-based system for detecting pathological myopia in ophthalmology training. By identifying knowledge gaps, providing personalized tutoring, and enhancing feedback, AI-assisted teaching methods may improve both teaching quality and learning outcomes (8–10).

With AI increasingly integrated into medical education, self-directed learning is crucial for medical students (11–13). Defined as an active process where learners set goals and manage their learning (14), self-directed learning is associated with academic performance (15, 16). Students with high self-directed learning skills achieve better results by actively engaging in learning, improving their goal-setting, resource access, and analysis skills (17). In medical education, where knowledge is rapidly updated and lifelong learning is essential, self-directed learning is particularly important for the development of independent professional competence (11, 18).

To examine these factors, this study integrates the Technology Acceptance Model (TAM) and the Unified Theory of Acceptance and Use of Technology 2 (UTAUT2). TAM provides a well-established foundation for examining how perceived usefulness and perceived ease of use shape learners’ behavioral intentions toward AI-assisted learning tools (19). However, as TAM was originally developed to explain individual-level technology acceptance (20), it does not readily account for the broader contextual and social factors that are likely to shape technology adoption in educational settings, such as social influence from peers and instructors and the availability of digital infrastructure. UTAUT2 addresses this limitation by incorporating these contextual constructs explicitly (21). In the context of self-directed learning, which involves both individual-level processes such as goal setting and self-monitoring and contextual conditions that shape how learners engage with learning tools (22), neither framework alone is sufficient; their integration therefore provides a more comprehensive theoretical basis for the present study. In the present study, TAM was used to represent technology-related beliefs, whereas UTAUT2 was used to represent broader contextual influences associated with AI-related behavioral intention.

In addition to technology-related and contextual perspectives, learner-related psychological factors such as motivation and attitude may also be relevant to self-directed learning in AI-assisted educational settings. Accordingly, this study aims to examine the learner-related, technology-related, and contextual factors associated with self-directed learning and AI-related behavioral intention among medical students in a medical university context. Understanding these associations may provide useful evidence for educators and institutions seeking to design AI-assisted learning environments that more effectively support students’ motivational engagement, self-directed learning processes, and technology adoption. Given the increasing integration of AI tools in health sciences education and the growing importance of self-directed learning competencies for future healthcare professionals, such evidence is particularly timely and relevant for informing curriculum design and instructional practice in this field.

### Supporting theory

#### Self-directed learning dimensions

Self-directed learning is both an effective pedagogy and a crucial competency, vital for improved educational outcomes and lifelong learning (23). Knowles defined it as a process where individuals, independently or with support, diagnose learning needs, set objectives, identify resources, implement strategies, and assess outcomes. Building on this, Ginzburg et al. (24) emphasized that self-directed learners must also identify, analyze, and synthesize relevant information, continuously self-regulate their learning, and embrace lifelong learning. Medical students must continually update their knowledge and engage in lifelong learning due to the life-critical nature of medicine (25). Self-directed learning is crucial for this lifelong engagement (26, 27), and improving these skills can boost motivation, reduce burnout, and ease teaching burdens. Self-directed learning is therefore a critical competency for medical students, and examining the learner-related and contextual factors associated with it is of clear importance in medical education.

#### Technology acceptance perspectives

The Technology Acceptance Model (TAM) explains how users come to accept and use new technologies. According to TAM, perceived ease of use and perceived usefulness shape behavioral intention, which in turn influences actual use behavior (19). External variables may also be associated with perceived ease of use and perceived usefulness, allowing researchers to incorporate additional factors relevant to technology use (28). TAM has been widely applied to examine learners’ acceptance of emerging technologies, including artificial intelligence in educational contexts (29, 30).

The Unified Theory of Acceptance and Use of Technology 2 (UTAUT2) also provides a useful perspective for understanding technology adoption (31). Its UTAUT2 has been shown to offer strong explanatory value in technology adoption research and highlights the importance of behavioral intention and contextual conditions in understanding technology use (21, 32). In the present study, TAM was used to represent technology-related beliefs, whereas UTAUT2 was used to represent broader contextual influences relevant to AI-related behavioral intention. Together, these perspectives provide a methodological basis for examining learner-related, technology-related, and contextual factors within the proposed conceptual framework.

#### Motivation and attitude

Recognizing the role of psychological factors, especially motivation and attitude, in shaping behavior, the model incorporates these constructs. Drawing on Bandhu’s motivation theory (33), in learning contexts, these factors are important for goal-directed behavior and may influence how learners regulate their learning. Therefore, motivation and attitude were included in the present study as key learner-related constructs.

## Method

### Conceptual framework, research questions, and hypotheses

**Figure 1:**
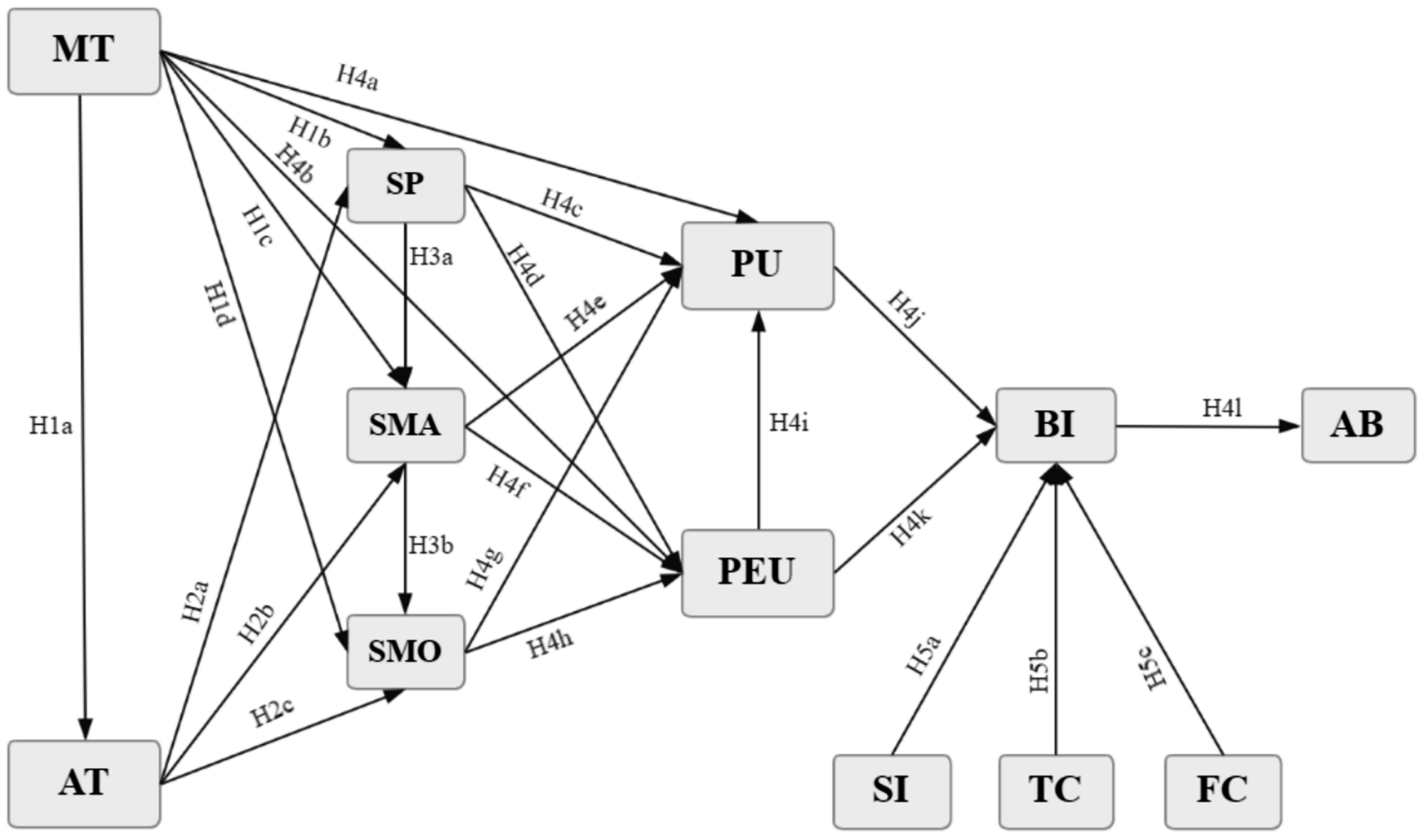
Integration of structural equation modeling. AT, attitude; MT, motivation; SP, self-planning; SMA, self-management; SMO, self-monitoring; PEU, perceived ease of use; PU, perceived usefulness; BI, behavior intention; AB, actual behavior, SI, social influence; TC, teacher characteristic; FC, facilitating condition.

This study developed a conceptual framework to examine self-directed learning among medical students in AI-assisted learning contexts. The framework draws on selected constructs from the Technology Acceptance Model (TAM), the Unified Theory of Acceptance and Use of Technology 2 (UTAUT2), motivational theory, and the literature on self-directed learning. Specifically, it incorporates learner-related, technology-related, and contextual variables to explain the hypothesized relationships shown in Figure 1. The study adopted a cross-sectional survey design, and all hypotheses were derived from established theoretical frameworks and tested in a confirmatory manner.

### Motivation

Self-determination theory, developed by Deci and Ryan, proposes that human behavior is voluntary and self-regulated (34). It also suggests that learning is more effective when driven by intrinsic motivation and supported by autonomy (35). Ntrinsic motivation includes internal factors such as curiosity, interest, and emotion, whereas extrinsic motivation is related to external rewards or reliance on specific techniques or tools (35). Prior literature has further emphasized the importance of motivation in self-directed learning (36). These theoretical perspectives support the inclusion of motivation as an important learner-related factor in self-directed learning. In this study, motivation refers to the psychological drive that encourages students to engage in self-directed learning. Accordingly, motivation was treated as a key learner-related construct in the conceptual framework.

*H1a*: Motivation positively influences attitude.

*H1b*: Motivation positively influences self-planning.

*H1c*: Motivation positively influences self-management.

*H1d*: motivation positively influences self-monitoring.

### Attitude

Attitude refers to learners’ evaluative orientation toward AI-assisted self-directed learning. Prior literature suggests that attitude is associated with how learners approach learning tasks and regulate their learning behaviors (35). In technology-enhanced learning contexts, students’attitudes may also be shaped by their perceptions of the learning environment (37). Accordingly, attitude was included in the present study as an important learner-related construct, and the following hypotheses were proposed:

*H2a*: Attitude positively influences self-planning.

*H2b*: Attitude positively influences self-management.

*H2c*: Attitude positively influences self-monitoring.

### Self-directed learning dimensions

Self-directed learning involves learners’ active regulation of their learning processes. Prior literature has described self-directed learning as involving planning, management, and monitoring of one’s own learning activities (38). In this study, self-planning refers to the organization of learning goals and activities, self-management refers to the regulation and adjustment of learning tasks, and self-monitoring refers to the ongoing review and evaluation of learning progress. These dimensions are conceptually related: effective self-planning may support self-management, and self-management may further facilitate self-monitoring. Accordingly, the following hypotheses were propose.

*H3a*: Self-planning has a positive influence on self-management.

*H3b*: Self-management positively influences self-monitoring.

### TAM model and UTAUT2 model

In the present study, perceived usefulness and perceived ease of use were included as core technology-related constructs derived from TAM. Perceived usefulness refers to the extent to which learners believe that using AI-related learning tools may enhance their learning, whereas perceived ease of use refers to the extent to which such tools are perceived as easy to use (39). Prior studies have suggested that learner-related factors may be associated with students’evaluations of learning technologies (40). Consistent with TAM, perceived ease of use was hypothesized to positively influence perceived usefulness, and both constructs were expected to positively influence behavioral intention, which in turn was expected to positively influence actual behavior (39). This study aligns with Davis’s framework by proposing the following hypotheses:

*H4a*: Motivation positively influences perceived usefulness.

*H4b*: Motivation positively influences perceived ease of use.

*H4c*: Self-planning positively influences perceived usefulness.

*H4d*: Self-planning positively influences perceived ease of use.

*H4e*: Self-management positively influences perceived usefulness.

*H4f*: Self-management positively influences perceived ease of use.

*H4g*: Self-monitoring positively influences perceived usefulness.

*H4h*: Self-monitoring positively influences perceived ease of use.

*H4i*: Perceived ease of use positively influences perceived usefulness.

*H4j*: Perceived usefulness positively influences behavioral intention.

*H4k*: Perceived ease of use positively influences behavioral intentions.

*H4l*: Behavioral intentions positively influences actual behavior.

### External environmental factors

In addition to learner-related and technology-related factors, contextual conditions may also shape students’ intention to use AI-related learning tools. In this study, social influence, teacher characteristics, and facilitating conditions were included as contextual variables. Social influence reflects perceived expectations or encouragement from important others, teacher characteristics reflect instructor-related factors relevant to AI-assisted learning, and facilitating conditions refer to the perceived availability of resources and support for technology use (21), Accordingly, the following hypotheses were proposed:

*H5a*: Social Influences Positively Influence Behavioral Intentions.

*H5b*: Teacher characteristics positively influence behavioral intentions.

*H5c*: Facilitating conditions positively influence behavioral intentions.

### Participant process

Before the formal survey, a pilot survey involving 151 students was conducted using an online questionnaire, and minor wording revisions were made to improve questionnaire clarity and quality. The formal survey adopted a cross-sectional design and a convenience sampling strategy. An online questionnaire was distributed via QR code in classrooms, small-group learning settings, and other offline contexts at a medical university. Participation was voluntary and anonymous. Eligible participants were students currently enrolled in undergraduate or postgraduate programs at the institution who provided informed consent prior to completion.

Data collection was conducted in three rounds. In the first round, 300 questionnaires were allocated for data quality assessment, and the recovery rate (91.3%) was satisfactory. In the second and third rounds, 300 and 228 questionnaires were collected respectively, bringing the total number of collected questionnaires to 828. For cases with one or two missing item responses within a construct, missing values were imputed using the mean value of the remaining items within the same construct prior to analysis. Questionnaires were excluded from analysis if they contained unanswered items (98 cases), were completed in an unreasonably short time suggesting insufficient engagement (72 cases), or contained logically inconsistent responses identified through attention check items (58 cases). A total of 600 valid responses were finally retained for analysis, yielding an effective response rate of 72.5%, which meets the ‘10-fold rule’ for the minimum sample size for structural equation modeling (41). Ethical approval was obtained from the Ethics Committee of Dalian Medical University (DMU-Ethics-2024-026). All procedures complied with the Declaration of Helsinki, and informed consent was obtained from all participants before questionnaire completion.

**Figure 2:**
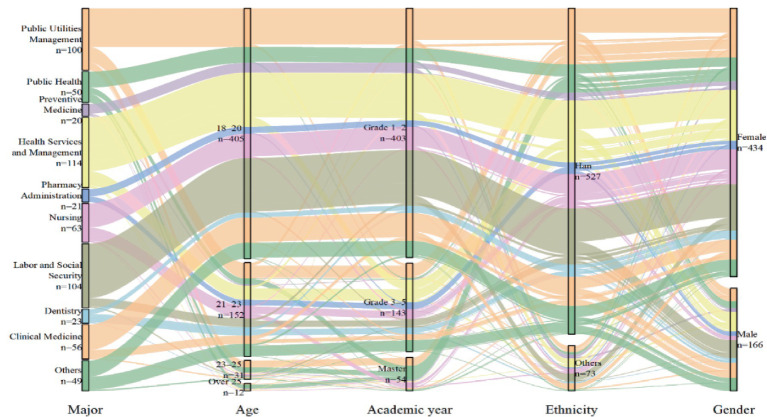
Demographic characteristics.

Based on the final sample of 600 valid questionnaires, the majority of participants were female (*n* = 434, 72.3%) and of Han ethnicity (*n* = 527, 87.8%). Most respondents were aged 18–20 years (*n* = 405, 67.5%), followed by those aged 21–23 years (*n* = 152, 25.3%). In terms of academic year, most participants were in grades 1–2 (*n* = 403, 67.2%), while 143 (23.8%) were in grades 3–5 and 54 (9.0%) were master’s students. Regarding majors, the largest proportion of participants were from Health Services and Management (*n* = 114, 19.0%), followed by Labor and Social Security (*n* = 104, 17.3%) and Public Utilities Management (*n* = 100, 16.7%). The characteristics of the participants are shown in Figure 2.

### Measurement

The research questionnaire comprised 47 items derived from previously validated instruments and adapted to align with the present study’s model and objectives (42–47), including the Self-Directed Learning Scale, the TAM questionnaire, and UTAUT-related items. The questionnaire was organized into two sections: the first section collected basic demographic information, and the second section contained the measurement items. Items were originally developed in English and translated into Chinese by the research team, with wording refined based on pilot survey feedback. All items were assessed using a five-point Likert scale ranging from 1 (“strongly disagree”) to 5 (“strongly agree”), with reverse-scored items recoded prior to analysis. The full item wording, construct–item mapping, and original sources are presented in Appendix 1.

### Data processing and analysis

Data were analyzed using a two-stage partial least squares structural equation modeling (PLS-SEM) procedure. Descriptive statistics and correlation analyses were conducted in SPSS 27, structural equation modeling was performed in SmartPLS 4, and all figures were generated in Origin 2024.

In the first stage, the reflective measurement model was evaluated. Internal consistency reliability was assessed using Cronbach’s alpha and composite reliability (CR), with values of 0.70 or higher considered acceptable. Convergent validity was assessed using indicator outer loadings and average variance extracted (AVE), with outer loadings generally expected to exceed 0.70 and AVE values to exceed 0.50 (48). Discriminant validity was assessed using both the Fornell–Larcker criterion and the heterotrait–monotrait ratio (HTMT) (49). In addition to HTMT point estimates, HTMT inference was conducted using bootstrapped confidence intervals, and discriminant validity was considered acceptable when the confidence interval did not include 1 (50).

In the second stage, the structural model was evaluated. Multicollinearity among predictor constructs was assessed using variance inflation factors (VIF), with values below 5 considered acceptable (48). Explanatory power was assessed using the coefficient of determination (R^2^), while effect sizes (f^2^) and predictive relevance (Q^2^) were also examined. Hypotheses were tested using bootstrapping with 5,000 resamples to estimate t-values and bias-corrected 95% confidence intervals for direct effects. Model fit was assessed using the standardized root mean square residual (SRMR), with values below 0.08 indicating acceptable fit. The normed fit index (NFI) was also reported as a supplementary fit index and interpreted cautiously (51).

## Results

### Measurement model assessment

We assessed the reliability and validity of the reflective measurement model. As shown in Appendix 2; Table 1, the indicator outer loadings ranged from 0.771 to 0.929, and all retained indicators were above the recommended threshold. Cronbach’s *α* values for the multi-item constructs ranged from 0.749 to 0.941, while composite reliability (CR) values ranged from 0.752 to 0.941. These results indicate satisfactory internal consistency reliability of the measurement model. In addition, the average variance extracted (AVE) values ranged from 0.666 to 0.855, all exceeding the recommended threshold of 0.50, thereby supporting convergent validity (Table 1).

**Table 1:** Questionnaire reliability and validity.

Endogenous constructs	Cronbach’s alpha	CR	AVE
SMO	0.749	0.752	0.666
SMA	0.776	0.777	0.690
TC	0.806	0.844	0.717
SP	0.815	0.818	0.730
SI	0.825	0.846	0.74
PEU	0.832	0.851	0.7501
MT	0.917	0.919	0.752
FC	0.844	0.844	0.763
PU	0.845	0.863	0.764
BI	0.875	0.876	0.800
AT	0.941	0.941	0.809
AB	0.915	0.92	0.855

**Figure 3:**
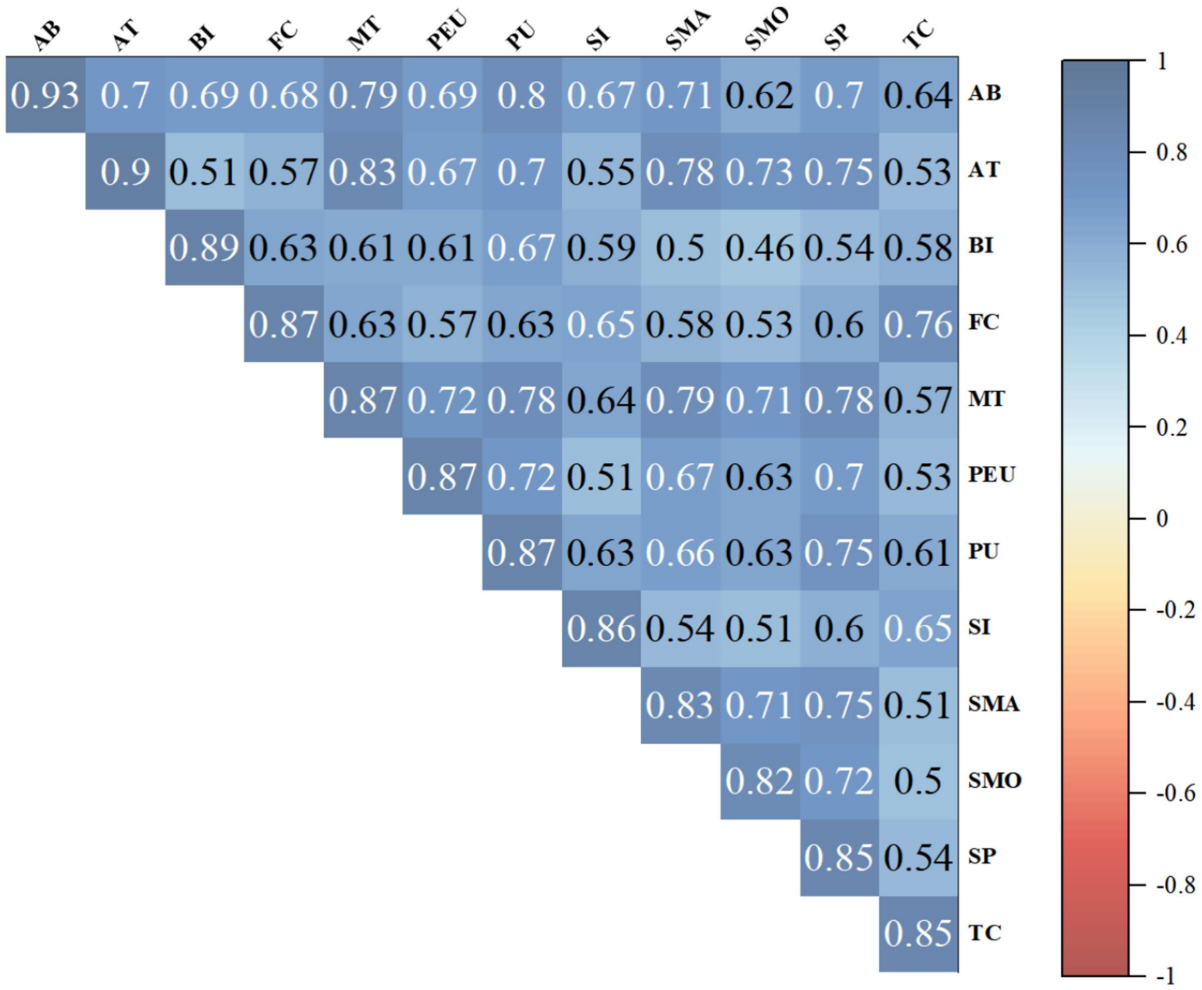
Correlation analysis.

Discriminant validity was subsequently assessed using both the Fornell–Larcker criterion and the heterotrait–monotrait ratio (HTMT). As presented in Figure 3, the square roots of the AVE values for all constructs were greater than their correlations with other constructs, indicating acceptable discriminant validity according to the Fornell–Larcker criterion. In addition, HTMT inference was conducted using bootstrap confidence intervals. All HTMT confidence intervals were below 1, further supporting discriminant validity. The detailed HTMT results are provided in Appendix 2; Table 2.

**Table 2:** Structural model quality (*R*^2^, *Q*^2^).

Endogenous	Q^2^ predict	R-square
AB	0.549	0.476
AT	0.693	0.695
BI	0.478	0.551
PEU	0.515	0.580
PU	0.606	0.685
SMA	0.626	0.700
SMO	0.493	0.588
SP	0.611	0.645

### Structural model assessment

Following the guidelines established by Hair et al. (41, 48), we conducted a comprehensive assessment of the structural model, focusing on collinearity diagnostics, model fit, explanatory power (R^2^), predictive relevance (Q^2^), and effect sizes (f^2^).

Initially, collinearity within the structural model was examined. The variance inflation factor (VIF) values for all predictor constructs ranged from 1.0 to 4.02. Although some predictors showed relatively elevated VIF values, all values remained below the threshold of 5, suggesting that multicollinearity was within an acceptable range.

Model fit was further assessed using the standardized root mean square residual (SRMR) and the normed fit index (NFI). The SRMR values were 0.053 for the saturated model and 0.110 for the estimated model, while the corresponding NFI values were 0.810 and 0.791, respectively. These results suggest that the saturated model showed acceptable fit, whereas the estimated model fit remained less than ideal and should therefore be interpreted with caution.

Regarding explanatory power, the model explained 47.6% of the variance in actual behavior, 69.5% in attitude, 55.1% in behavioral intention, 58.0% in perceived ease of use, 68.5% in perceived usefulness, 70.0% in self-management, 58.8% in self-monitoring, and 64.5% in self-planning. These R^2^ values indicate moderate to substantial explanatory power overall. In addition, all Q^2^ values were greater than zero, supporting the predictive relevance of the model (see Table 2).

The effect size estimates (f^2^) varied considerably across the structural paths, and were interpreted according to Cohen’s (65) benchmarks: f^2^ ≥ 0.35 indicates a large effect, f^2^ ≥ 0.15 a medium effect, f^2^ ≥ 0.02 a small effect, and f^2^ < 0.02 a negligible effect. A large effect was observed for the relationship between behavioral intention and actual behavior (f^2^ = 0.908). Motivation also showed a relatively strong effect on attitude (f^2^ = 2.281), although this result should be interpreted with caution given the magnitude of the estimate. A moderate effect was found for motivation on self-planning (f^2^ = 0.227). Most of the remaining paths showed only small or negligible effect sizes, as detailed in Table 3.

**Table 3:** Effect sizes (*f*^2^) for path.

Path	f-square	Effect size
MT → AT	2.281	Large
BI → AB	0.908	Large
MT → SP	0.226	Medium
MT → PU	0.137	Small
AT → SMA	0.094	Small
AT → SP	0.090	Small
MT → SMA	0.089	Small
PEU → PU	0.084	Small
AT → SMO	0.080	Small
MT → PEU	0.078	Small
SP → SMA	0.076	Small
SP → PU	0.070	Small
SMA → SMO	0.061	Small
PU → BI	0.060	Small
SP → PEU	0.043	Small
PEU → BI	0.038	Small
FC → BI	0.030	Small
SI → BI	0.027	Small
MT → SMO	0.022	Small
SMO → PEU	0.015	Negligible
SMA → PEU	0.012	Negligible
SMA → PU	0.003	Negligible
TC → BI	0.003	Negligible
SMO → PU	0.001	Negligible

### Path factor

Regarding the direct structural paths, the results provided support for the proposed model structure. Specific path coefficients, T-statistics, and significance levels are summarized in Table 4.

For H1 and H2, motivation emerged as the most dominant antecedent, showing a large effect on attitude (*β* = 0.834, *p* < 0.001, f^2^ = 2.281) and significant positive associations with all three self-directed learning dimensions (H1b–H1d: all *p* < 0.05). Attitude further demonstrated significant positive associations with all three self-directed learning dimensions (H2a–H2c: all *p* < 0.01), supporting H2a through H2c.

Regarding H3, self-planning significantly predicted self-management (*β* = 0.253, *p* < 0.001), and self-management in turn significantly influenced self-monitoring (*β* = 0.280, *p* < 0.001), confirming a sequential pattern among these components and supporting H3a and H3b.

For the TAM and UTAUT related paths (H4), motivation and self-planning significantly predicted both perceived usefulness and perceived ease of use (H4a–H4d: all *p* < 0.001). Self-management showed a non-significant negative association with perceived usefulness (H4e: *β* = −0.052, *p* = 0.204), and self-monitoring showed a non-significant association with perceived usefulness (H4g: *β* = 0.029, *p* = 0.310), leading to the rejection of H4e and H4g. Self-management and self-monitoring both showed significant positive associations with perceived ease of use (H4f: *p* < 0.05; H4h: p < 0.05). Perceived ease of use significantly predicted perceived usefulness (H4i: *β* = 0.251, *p* < 0.001), and both perceived usefulness and perceived ease of use significantly predicted behavioral intention (H4j–H4k: all *p* < 0.001). Behavioral intention showed a large positive association with actual behavior (H4l: *β* = 0.690, *p* < 0.001, f^2^ = 0.908).

Among the external environmental factors, social influence (H5a: *β* = 0.160, *p* < 0.01) and facilitating conditions (H5c: *β* = 0.196, *p* < 0.001) both significantly predicted behavioral intention, whereas teacher characteristics did not reach significance (H5b: *β* = 0.064, *p* = 0.139), leading to the rejection of H5b.

**Table 4:** Path coefficients and significance testing results.

Hypotheses	Path	β	T statistics	*p* values
H1a	MT → AT	0.834	37.608	<0.001
H1b	MT → SP	0.514	4.952	<0.001
H1c	MT → SMA	0.328	5.392	<0.001
H1d	MT → SMO	0.188	2.207	<0.05
H2a	AT → SP	0.323	3.230	<0.01
H2b	AT → SMA	0.318	4.796	<0.001
H2c	AT → SMO	0.353	4.820	<0.001
H3a	SP → SMA	0.253	5.520	<0.001
H3b	SMA → SMO	0.280	4.835	<0.001
H4a	MT → PU	0.406	4.422	<0.001
H4b	MT → PEU	0.341	4.787	<0.001
H4c	SP → PU	0.273	3.914	<0.001
H4d	SP → PEU	0.240	3.653	<0.001
H4e	SMA → PU	−0.052	0.827	0.204
H4f	SMA → PEU	0.130	1.854	<0.05
H4g	SMO → PU	0.029	0.496	0.310
H4h	SMO → PEU	0.126	2.283	<0.05
H4i	PEU → PU	0.251	3.877	<0.001
H4j	PU → BI	0.270	5.315	<0.001
H4k	PEU → BI	0.191	3.990	<0.001
H4l	BI → AB	0.690	24.11	<0.001
H5a	SI → BI	0.160	2.921	<0.01
H5b	TC → BI	0.064	1.084	0.139
H5c	FC → BI	0.196	3.873	<0.001

## Discussion

This study tested an integrated conceptual framework drawing on TAM, UTAUT2, and self-directed learning literature to examine how learner-related, technology-related, and contextual factors were associated with self-directed learning and AI-related behavioral intention among medical students at a medical university. The results supported 21 of the 24 hypothesized paths. Three non-significant findings, H4e (SMA to PU), H4g (SMO to PU), and H5b (TC to BI), are discussed alongside the supported paths, as they may be as informative as the confirmed associations.

### Motivation

Motivation emerged as the strongest predictor of attitude and showed significant direct associations with all three self-directed learning dimensions, providing support for all paths in H1. This pattern is consistent with self-determination theory’s position that autonomous motivation is a key precondition for self-directed learning engagement, and with evidence from a randomized controlled trial in medical education showing that autonomy-supportive teaching is associated with improved intrinsic motivation among medical students (52). The parallel structure observed here, whereby motivation was associated with self-directed learning dimensions both directly and through attitude, suggests that motivational resources may not be fully channeled through a single pathway, though the cross-sectional design limits any causal interpretation of this pattern.

These findings suggest that motivational orientation may be a relevant consideration in the design of AI-assisted learning environments for medical students. Whether explicitly supporting students’ motivation, such as through adaptive feedback mechanisms within AI platforms, would translate into stronger self-directed learning outcomes is a question that future interventional research would need to address. It is also worth noting that motivational engagement with AI tools does not necessarily translate into deep or independent learning. Medical students who are motivated primarily by efficiency may use AI to generate answers rather than to support independent reasoning, a pattern consistent with evidence that excessive reliance on AI-generated content may lead to cognitive inertia and a reduced capacity for independent thought among medical students (53). This consideration suggests that motivational support in AI-assisted environments should be accompanied by pedagogical guidance on how to engage with AI tools in ways that promote rather than substitute independent cognitive effort.

### Attitude

Attitude significantly predicted all three self-directed learning dimensions, providing support for H2. This pattern is broadly consistent with TAM-based evidence that attitudinal disposition toward technology is associated with subsequent behavioral engagement (54), though it should be noted that Ibrahim et al. examined general adult samples rather than medical students, and the present interpretation, that attitude may be associated with SDL behaviors through its role as a linking construct between motivation and self-regulatory engagement, is proposed on the basis of the current data rather than directly established by prior work.

The nature of medical students’ attitudinal orientation toward AI-assisted learning may also be worth attending to. Medical students have been found to express both enthusiasm and perceived barriers toward AI use in learning, including concerns about reliability and negative impacts on critical thinking, with confusion arising partly from contradictory messages received from educators (55). Evidence from medical education contexts indicates that excessive reliance on AI may foster intellectual passivity, undermine higher-order reasoning skills, and raise academic integrity concerns (56), further supporting the view that a balanced and critically informed attitude toward AI may be more educationally productive than uncritical acceptance. Given the increasing use of AI technologies such as ChatGPT in education, cultivating this kind of informed and critical attitude may be as important as promoting technology acceptance itself. A cross-sectional study in higher education found that technology readiness and social influence were positively associated with the perceived usefulness and effectiveness of AI-assisted learning systems, highlighting the need for targeted policies and evidence-based strategies to support effective and responsible implementation (57).

### Self-directed learning dimensions

The significant paths from self-planning to self-management and from self-management to self-monitoring provide support for H3 and suggest that these self-directed learning components may be organized sequentially in the present sample. This sequential pattern is theoretically consistent with Garrison’s model of self-directed learning, in which planning is conceptualized as an initiating phase that structures subsequent management and monitoring of learning activities This theoretical sequence has been summarized in recent literature on digital health education (58), though that work draws on conceptual rather than empirical health sciences student data. Whether the observed sequence reflects a causal developmental structure cannot be determined from cross-sectional data.

One of the more unexpected patterns in the data is that self-planning was associated with both perceived usefulness and perceived ease of use (H4c, H4d supported), while self-management was not associated with perceived usefulness (H4e not supported) but showed a significant positive association with perceived ease of use (H4f supported), and self-monitoring was associated with perceived ease of use (H4h supported) but not with perceived usefulness (H4g not supported). This asymmetric pattern suggests that self-directed learning dimensions may not relate to technology acceptance beliefs in a uniform way. One possible interpretation is that goal-directed planning, as a cognitively structured activity oriented toward learning objectives, may provide a frame through which medical students evaluate whether a technology is likely to be useful and accessible for their purposes. Self-management and self-monitoring, which operate at more task-specific behavioral and evaluative levels, may be less directly connected to broader evaluative beliefs about technology usefulness, though they may still relate to perceptions of ease of use. This interpretation is proposed tentatively and should be treated with caution, particularly given that several HTMT values in the measurement model approached boundary thresholds, which introduces some uncertainty about the precision of the sub-dimension constructs. The pattern warrants replication before stronger conclusions are drawn.

It is also worth considering whether AI-assisted learning environments may differentially affect these dimensions. While AI tools may support the planning and organization of learning activities, medical students who rely on AI-generated feedback as a substitute for independent self-evaluation may experience a reduced demand for active self-monitoring. Evidence suggests that over-reliance on AI dialogue systems can diminish students’ engagement with self-directed learning processes, including monitoring and evaluating their own learning progress (59). Whether AI assistance strengthens or attenuates self-monitoring behaviors among medical students is a question that future longitudinal research should examine.

If the sequential self-directed learning pattern proves stable in future longitudinal work, it would suggest that planning-focused activities may be a productive starting point for self-directed learning development in AI-assisted curricula, with potential downstream effects on management and monitoring. This remains a hypothesis for future research.

### TAM model and UTAUT2 model

The paths from perceived ease of use to perceived usefulness and to behavioral intention, and from behavioral intention to actual behavior (H4i–H4l), were all significant, which is consistent with the core TAM structure. In a large-sample study of AI adoption among general adult users, perceived usefulness and ease of use were confirmed as significant predictors of attitude and behavioral intention (54), This is broadly consistent with findings from medical education contexts suggesting that medical students’ perceptions of AI tools are shaped by both individual and contextual factors (64).

### External environmental factors

Among the external constructs, social influence and facilitating conditions were both associated with behavioral intention (H5a, H5c supported), while teacher characteristics were not (H5b not supported). This pattern may partly reflect the composition of the present sample, which consisted predominantly of public health and health management students whose learning context may involve less reliance on individual instructor relationships compared with clinical training programs. This interpretation is speculative, however, and alternative explanations, including limitations in how teacher characteristics were operationalized in this study, cannot be ruled out.

The significance of facilitating conditions points to a practical consideration: access to digital infrastructure may be a relevant boundary condition for AI-related behavioral intention in this population. Among university educators in Arab countries, technology readiness and social influence were found to be associated with perceived AI usefulness (57); while that study examined educators rather than students, it suggests that contextual and infrastructure-related factors may be relevant to technology acceptance across higher education populations. Importantly, however, improved access to AI tools does not necessarily translate into educationally beneficial use. Evidence from medical education contexts indicates that limited digital infrastructure, internet connectivity, and data storage capacity can restrict equitable AI adoption among medical students, particularly in resource-constrained settings (63). A review of AI integration in pre-clinical medical education further noted that ensuring equal and fair access to AI tools for all students is crucial to avoid disparities in educational opportunities (60). Medical education institutions should therefore consider not only providing digital infrastructure but also supporting medical students in developing the critical skills and ethical awareness needed to engage with AI tools responsibly and equitably.

### Theoretical and practical implications

The present findings extend prior work by suggesting that motivation and attitude may function as relevant antecedents of SDL engagement in AI-assisted health sciences education, and that the association between SDL sub-dimensions and technology acceptance beliefs may not be uniform across sub-dimensions. These patterns, if replicated, may offer a useful lens for understanding how self-regulatory processes and technology acceptance interact in this context. The integrated SDL–TAM–UTAUT2 approach appears to capture associations that neither framework alone would readily reveal, though this observation is based on a single cross-sectional study and should be treated accordingly.

From a practical standpoint, the findings suggest several directions worth exploring in future work. If motivational orientation is associated with SDL engagement, assessing and supporting students’ motivation alongside AI tool deployment may be a worthwhile component of instructional design. The sequential SDL pattern suggests that planning-focused activities may be a productive starting point for SDL development. Regarding teacher characteristics, the non-significant association with behavioral intention raises the possibility that instructors’ contribution in AI-assisted environments may be better directed toward areas that technology cannot replicate, such as facilitating critical evaluation of AI-generated content and supporting professional judgment development, though this is an interpretation of the present data rather than a finding. Recent practical guidance for medical educators similarly emphasizes that AI tools should complement rather than replace the development of professional judgment and ethical awareness in medical training (61).

### Study limitations and future recommendations

Several limitations should be considered when interpreting the present findings. First, the cross-sectional design precludes causal inference; the associations identified in the structural model cannot establish temporal directionality or exclude the possibility of unmeasured confounding. Second, the sample was drawn from a single medical university using a convenience sampling strategy, which limits the generalizability of the findings. The sample also showed a notable gender imbalance, with approximately 72.3% female participants. In addition, subgroup sensitivity analyses by gender or academic program were not conducted, and broader generalization will therefore require more diverse, multi-institutional samples. Third, measurement-related limitations should also be acknowledged. All variables were assessed using self-administered questionnaires, introducing susceptibility to self-report bias and common method variance, while the absence of objective outcome indicators means that the behavioral constructs reflect perceived rather than directly observed conduct. Furthermore, several HTMT results were close to the decision boundary, and the SRMR value did not fully satisfy conventional thresholds, both of which should be taken into account when evaluating the strength of the conclusions. Finally, the study did not capture potentially relevant sociodemographic and contextual variables, including household economic status, internet access quality, and urban–rural location, which may represent important explanatory factors in students’ engagement with AI-assisted learning.

Future research should extend the present findings by addressing both substantive and methodological limitations. In substantive terms, greater attention should be given to the ethical and contextual dimensions of AI-assisted learning, including data privacy, academic integrity, and equity of access, as these factors may shape medical students’ self-directed learning, engagement, trust, and the effective adoption of AI technologies in educational settings (62). In methodological terms, longitudinal and multi-institutional study designs are needed to determine whether the associations identified in the present study remain stable across different student populations and educational contexts, and to clarify the temporal ordering of the observed relationships.

## Conclusion

This study suggests that self-directed learning and AI-related behavioral intention among medical students are associated with both learner-related and contextual factors in AI-assisted learning environments. Motivation emerged as the strongest predictor in the model, showing a large effect on attitude and significant positive associations with all three self-directed learning dimensions. Behavioral intention also showed a large positive association with actual behavior. A sequential pattern was observed among the self-directed learning dimensions, whereby self-planning was associated with self-management, and self-management was in turn associated with self-monitoring. Among the technology acceptance constructs, perceived ease of use and perceived usefulness were both significantly associated with behavioral intention, consistent with the core TAM structure. Among the contextual factors, facilitating conditions and social influence were significantly associated with behavioral intention, whereas teacher characteristics were not. Self-management and self-monitoring were not significantly associated with perceived usefulness, suggesting that the relationship between self-directed learning dimensions and technology acceptance beliefs may not be uniform across sub-dimensions.

These findings suggest that AI integration in health sciences education should place greater emphasis on students’ motivational orientation, planning-related learning activities, and supportive learning conditions. In addition to fostering constructive attitudes toward AI-assisted learning, institutions may need to strengthen motivational support, structured opportunities for self-directed planning, and adequate digital infrastructure.

Overall, this study provides preliminary evidence that integrating self-directed learning dimensions with TAM and UTAUT related constructs may help explain AI-assisted learning behavior in health sciences education. Longitudinal and multi-institutional research is needed to examine whether these associations are consistent across different student populations and educational contexts.

## Data Availability

The raw data supporting the conclusions of this article will be made available by the authors, without undue reservation.
