# Supplementary material for: Examining the association between AI-enhanced education and medical students’ self-directed learning using an integrated TAM-UTAUT2 model
Source: Front Med (Lausanne). 2026 Apr 24;13:1817255. doi: 10.3389/fmed.2026.1817255 (PMC13154930; doi:10.3389/fmed.2026.1817255)
Supplement: Supplementary file 1 [file Table_1.docx]

# Appendix 1

Part 1: Construct-Item Mapping and Original Sources

| Attitude (AT) | (Cadorin et al.,2013)( Cadorin et al.,2016) |
| --- | --- |
| Motivation (MT) | (Cheng et al.,2010)( Cadorin et al.,2013) |
| Self-planning (SP) |  |
| Self-management (SMA) |  |
| Self-monitoring (SMO) |  |
| Perceived Usefulness (PU) | (Chau,1996)(Zuo et al.,2022) |
| Perceived Ease of Use (PEU) |  |
| Behavioral Intention (BI) | (Venkatesh et al.,2012)( Prasetyo et al.,2021) |
| Actual Behavior (AB) | (Chau,1996)(Prasetyo et al.,2021) |
| Social Influence (SI) | ( Venkatesh et al.,2012)( Prasetyo et al.,2021)( Zuo et al.,2022) |
| Teacher Characteristics (TC) | (Shea,2007)( Zuo et al.,2022) |
| Facilitating Conditions (FC) | ( Venkatesh et al.,2012)( Prasetyo et al.,2021) |

**References**

Cadorin L, Bortoluzzi G, Palese A.The self-rating scale of self-directed learning (SRSSDL): a factor analysis of the Italian version[J]. Nurse Education Today, 2013, 33(12): 1511-1516. https://doi.org/10.1016/j.nedt.2013.04.010

Cadorin L,Cheng S F,Palese A.Concurrent validity of self-rating scale of self-directed learning and self-directed learning instrument among Italian nursing students[J].BMC nursing,2016,15(1):1-10.DOI:10.1186/s12912-016-0142-x

Cheng S F,Kuo C L,Lin K C,et al.Development and preliminary testing of a self-rating instrument to measure self-directed learning ability of nursing students[J]. International journal of nursing studies,2010,47(9): 1152-1158.https://doi.org/10.1016/j.ijnurstu.2010.02.002

Chau P Y K.An empirical assessment of a modified technology acceptance model[J].Journal of management information systems,1996,13(2): 185-204.DOI:10.1080/07421222.1996.11518128

Zuo M,Hu Y,Luo H,et al.K-12 students’online learning motivation in China:An integrated model based on community of inquiry and technology acceptance theory[J].Education and Information Technologies, 2022, 27(4):4599-4620. https://doi.org/10.1007/s10639-021-10791-x

Venkatesh V, Thong J Y L, Xu X. Consumer acceptance and use of information technology: extending the unified theory of acceptance and use of technology[J]. MIS quarterly, 2012: 157-178. https://doi.org/10.2307/41410412

Prasetyo Y T, Roque R A C, Chuenyindee T, et al. Determining factors affecting the acceptance of medical education elearning platforms during the covid-19 pandemic in the philippines: Utaut2 approach[C]//Healthcare. MDPI, 2021, 9(7): 780. https://doi.org/10.3390/healthcare9070780

Shea P. BRIDGES AND BARRIERS TO TEACHING ONLINE COLLEGE COURSES: A STUDY OF EXPERIENCED ONLINE FACULTY IN THIRTYSIX COLLEGES[J]. Journal of Asynchronous Learning Networks, 2007, 11(2). DOI:10.24059/olj.v11i2.1728

# Part 2: Questionnaire Items

# Questionnaire on Learning Modes in Medical Education

Comparing Traditional Learning and AI-Assisted Learning

**Dear Student,**

We are conducting a study on learning modes in medical education, aiming to compare the effectiveness of traditional learning methods and AI-assisted learning methods. This questionnaire is anonymous, and your responses will be used only for academic research. We will strictly maintain confidentiality. The questionnaire takes approximately 10 minutes to complete. Thank you for your support!

## Part 1: Basic Information

1. Gender: ☐ Male ☐ Female

2. Grade: _____________

3. Major: ☐ Clinical Medicine ☐ Nursing ☐ Other: _______

4. Education Level: _____________

5. Ethnicity: _____________

## Part 2: Assessment of Learning Attitudes and Behaviors

**Rating Instructions:** Please rate based on your actual experience in both learning stages (Traditional Learning and AI-assisted Learning).

**Rating Scale:** 5 = Strongly Agree | 4 = Agree | 3 = Neutral | 2 = Disagree | 1 = Strongly Disagree

| **Attitude** |  |  |  |  |  |
| --- | --- | --- | --- | --- | --- |
| I will study seriously | **5** | **4** | **3** | **2** | **1** |
| I am well aware of the knowledge points I need to learn | **5** | **4** | **3** | **2** | **1** |
| As time goes by, I can maintain my learning motivation | **5** | **4** | **3** | **2** | **1** |
| I can plan and define my learning goals | **5** | **4** | **3** | **2** | **1** |
| I can choose the learning method that suits me best | **5** | **4** | **3** | **2** | **1** |
| **Motivation** |  |  |  |  |  |
| I know what I need to learn | **5** | **4** | **3** | **2** | **1** |
| Regardless of the results or effectiveness of my learning, I still enjoy learning. | **5** | **4** | **3** | **2** | **1** |
| I hope my grades can continuously improve during the learning process. | **5** | **4** | **3** | **2** | **1** |
| My success or failure in learning motivates me to continue learning | **5** | **4** | **3** | **2** | **1** |
| I like to find answers to questions | **5** | **4** | **3** | **2** | **1** |
| **Self-management** |  |  |  |  |  |
|  |  |  |  |  |  |
| I know what learning strategies suit me | **5** | **4** | **3** | **2** | **1** |
| Whether in clinical practice, after class, or self-study, I can follow my learning plan | **5** | **4** | **3** | **2** | **1** |
| I am good at arranging and controlling my learning time | **5** | **4** | **3** | **2** | **1** |
| **Self-monitoring** |  |  |  |  |  |
|  |  |  |  |  |  |
| I can monitor my learning progress | **5** | **4** | **3** | **2** | **1** |
| I can evaluate my learning outcomes by myself | **5** | **4** | **3** | **2** | **1** |
| I will carefully understand my learning process | **5** | **4** | **3** | **2** | **1** |
| **Self-planning** |  |  |  |  |  |
|  |  |  |  |  |  |
| I can actively establish my learning goals | **5** | **4** | **3** | **2** | **1** |
| I have set priorities for my learning | **5** | **4** | **3** | **2** | **1** |
|  |  |  |  |  |  |
| I know how to find resources for my learning | **5** | **4** | **3** | **2** | **1** |
| **Perceived usefulness** |  |  |  |  |  |
| Without self-directed learning, my learning would be difficult to carry out effectively | **5** | **4** | **3** | **2** | **1** |
| Self-directed learning improves my learning efficiency | **5** | **4** | **3** | **2** | **1** |
| Self-directed learning is very useful for my learning | **5** | **4** | **3** | **2** | **1** |
| **Perceived ease of use** |  |  |  |  |  |
| Self-directed learning is easy to conduct | **5** | **4** | **3** | **2** | **1** |
| I can easily understand knowledge related to self-directed learning | **5** | **4** | **3** | **2** | **1** |
| When conducting self-directed learning, I find the process difficult | **5** | **4** | **3** | **2** | **1** |
| **Social influence** |  |  |  |  |  |
| People around me (parents, teachers, classmates, friends, etc.) think I should engage in self-directed learning | **5** | **4** | **3** | **2** | **1** |
| The surrounding environment affects my efficiency in self-directed learning | **5** | **4** | **3** | **2** | **1** |
| The surrounding environment stimulates my desire to engage in self-directed learning | **5** | **4** | **3** | **2** | **1** |
| **Behavioral intentions** |  |  |  |  |  |
| I plan to continue self-directed learning in the near future | **5** | **4** | **3** | **2** | **1** |
| I will always try to engage in self-directed learning in daily life | **5** | **4** | **3** | **2** | **1** |
| I plan to frequently engage in self-directed learning | **5** | **4** | **3** | **2** | **1** |
| **Actual behavior** |  |  |  |  |  |
|  |  |  |  |  |  |
| I frequently engage in self-directed learning during my studies | **5** | **4** | **3** | **2** | **1** |
| I rely on self-directed learning | **5** | **4** | **3** | **2** | **1** |
| Self-directed learning serves as my main learning tool | **5** | **4** | **3** | **2** | **1** |
| **Teacher characteristics** |  |  |  |  |  |
| Teachers encourage us to engage in self-directed learning | **5** | **4** | **3** | **2** | **1** |
| I am invited by teachers to ask questions/get answers in class | **5** | **4** | **3** | **2** | **1** |
| Teachers supervise me in self-directed learning | **5** | **4** | **3** | **2** | **1** |
| **Facilitating conditions** |  |  |  |  |  |
| Self-directed learning provides convenience for me in learning | **5** | **4** | **3** | **2** | **1** |
| I have the conditions to engage in self-directed learning | **5** | **4** | **3** | **2** | **1** |
| When encountering difficulties in self-directed learning, specific people (or groups) are available to help | **5** | **4** | **3** | **2** | **1** |

**End of questionnaire. Thank you for your participation!**
