# Supplementary material for: Examining the association between AI-enhanced education and medical students’ self-directed learning using an integrated TAM-UTAUT2 model
Source: Front Med (Lausanne). 2026 Apr 24;13:1817255. doi: 10.3389/fmed.2026.1817255 (PMC13154930; doi:10.3389/fmed.2026.1817255)
Supplement: Supplementary file 2 [file Table_2.docx]

Appendix 2

Table 1: Indicator Outer Loadings

| Item | Outer loadings |
| --- | --- |
| AB1 <- AB | 0.926 |
| AB2 <- AB | 0.919 |
| AB3 <- AB | 0.929 |
| AT1 <- AT | 0.907 |
| AT2 <- AT | 0.909 |
| AT3 <- AT | 0.898 |
| AT4 <- AT | 0.909 |
| AT5 <- AT | 0.875 |
| BI1 <- BI | 0.911 |
| BI2 <- BI | 0.887 |
| BI3 <- BI | 0.885 |
| FC1 <- FC | 0.875 |
| FC2 <- FC | 0.870 |
| FC3 <- FC | 0.876 |
| MT1 <- MT | 0.871 |
| MT2 <- MT | 0.830 |
| MT3 <- MT | 0.841 |
| MT4 <- MT | 0.897 |
| MT5 <- MT | 0.894 |
| PEU1 <- PEU | 0.902 |
| PEU2 <- PEU | 0.919 |
| PEU3 <- PEU | 0.771 |
| PU1 <- PU | 0.821 |
| PU2 <- PU | 0.916 |
| PU3 <- PU | 0.883 |
| SI1 <- SI | 0.857 |
| SI2 <- SI | 0.821 |
| SI3 <- SI | 0.902 |
| SMA1 <- SMA | 0.815 |
| SMA2 <- SMA | 0.854 |
| SMA3 <- SMA | 0.824 |
| SMO1 <- SMO | 0.791 |
| SMO2 <- SMO | 0.833 |
| SMO3 <- SMO | 0.824 |
| SP1 <- SP | 0.849 |
| SP2 <- SP | 0.884 |
| SP3 <- SP | 0.830 |
| TC1 <- TC | 0.883 |
| TC2 <- TC | 0.818 |
| TC3 <- TC | 0.837 |

Table 2 HTMT

| Path | Original sample (O) | Sample mean (M) | 5.0% | 95.0% |
| --- | --- | --- | --- | --- |
| BI <-> AT | 0.555 | 0.555 | 0.472 | 0.628 |
| SMO <-> BI | 0.567 | 0.567 | 0.481 | 0.646 |
| TC <-> AT | 0.603 | 0.602 | 0.528 | 0.672 |
| SMA <-> BI | 0.607 | 0.606 | 0.522 | 0.681 |
| SI <-> PEU | 0.610 | 0.610 | 0.527 | 0.690 |
| SI <-> AT | 0.618 | 0.618 | 0.540 | 0.689 |
| SP <-> BI | 0.633 | 0.632 | 0.555 | 0.701 |
| TC <-> SMO | 0.633 | 0.632 | 0.547 | 0.712 |
| TC <-> PEU | 0.635 | 0.635 | 0.553 | 0.713 |
| TC <-> SMA | 0.641 | 0.640 | 0.558 | 0.719 |
| FC <-> AT | 0.641 | 0.639 | 0.578 | 0.699 |
| SMO <-> SI | 0.643 | 0.643 | 0.553 | 0.725 |
| TC <-> SP | 0.646 | 0.646 | 0.559 | 0.728 |
| TC <-> MT | 0.651 | 0.650 | 0.582 | 0.714 |
| SMA <-> SI | 0.657 | 0.657 | 0.578 | 0.730 |
| SMO <-> FC | 0.668 | 0.667 | 0.596 | 0.734 |
| TC <-> BI | 0.669 | 0.669 | 0.591 | 0.742 |
| PEU <-> FC | 0.674 | 0.674 | 0.600 | 0.742 |
| MT <-> BI | 0.678 | 0.677 | 0.615 | 0.734 |
| SI <-> BI | 0.689 | 0.687 | 0.623 | 0.747 |
| PEU <-> BI | 0.707 | 0.707 | 0.630 | 0.780 |
| TC <-> PU | 0.711 | 0.711 | 0.651 | 0.769 |
| SMA <-> FC | 0.713 | 0.711 | 0.649 | 0.771 |
| SP <-> SI | 0.715 | 0.715 | 0.644 | 0.779 |
| MT <-> FC | 0.721 | 0.720 | 0.668 | 0.771 |
| SI <-> MT | 0.722 | 0.721 | 0.663 | 0.776 |
| SP <-> FC | 0.726 | 0.725 | 0.667 | 0.780 |
| FC <-> BI | 0.727 | 0.726 | 0.667 | 0.782 |
| TC <-> AB | 0.727 | 0.726 | 0.653 | 0.790 |
| PU <-> FC | 0.736 | 0.735 | 0.679 | 0.789 |
| SMO <-> AB | 0.738 | 0.739 | 0.649 | 0.818 |
| SI <-> PU | 0.741 | 0.740 | 0.671 | 0.805 |
| AT <-> AB | 0.754 | 0.754 | 0.681 | 0.818 |
| SI <-> AB | 0.754 | 0.754 | 0.699 | 0.805 |
| PEU <-> AT | 0.758 | 0.759 | 0.701 | 0.812 |
| TC <-> SI | 0.766 | 0.766 | 0.690 | 0.834 |
| BI <-> AB | 0.767 | 0.766 | 0.716 | 0.812 |
| PU <-> BI | 0.770 | 0.770 | 0.715 | 0.818 |
| SI <-> FC | 0.770 | 0.770 | 0.708 | 0.828 |
| FC <-> AB | 0.775 | 0.774 | 0.726 | 0.820 |
| PU <-> AT | 0.781 | 0.781 | 0.711 | 0.842 |
| SMO <-> PU | 0.784 | 0.784 | 0.704 | 0.856 |
| PEU <-> AB | 0.790 | 0.790 | 0.736 | 0.841 |
| SMO <-> PEU | 0.793 | 0.794 | 0.742 | 0.843 |
| SP <-> AB | 0.801 | 0.802 | 0.723 | 0.868 |
| SMA <-> PU | 0.809 | 0.808 | 0.735 | 0.873 |
| PEU <-> MT | 0.825 | 0.825 | 0.774 | 0.872 |
| SMA <-> PEU | 0.831 | 0.832 | 0.778 | 0.884 |
| SMA <-> AB | 0.834 | 0.834 | 0.783 | 0.880 |
| SP <-> PEU | 0.840 | 0.842 | 0.782 | 0.896 |
| SMO <-> MT | 0.845 | 0.845 | 0.784 | 0.896 |
| PU <-> PEU | 0.846 | 0.847 | 0.758 | 0.926 |
| MT <-> AB | 0.853 | 0.853 | 0.816 | 0.888 |
| SP <-> AT | 0.857 | 0.858 | 0.814 | 0.898 |
| SMO <-> AT | 0.869 | 0.869 | 0.830 | 0.907 |
| PU <-> MT | 0.879 | 0.879 | 0.828 | 0.922 |
| SP <-> PU | 0.893 | 0.893 | 0.808 | 0.967 |
| MT <-> AT | 0.894 | 0.895 | 0.857 | 0.928 |
| PU <-> AB | 0.900 | 0.900 | 0.857 | 0.939 |
| SP <-> MT | 0.903 | 0.904 | 0.840 | 0.955 |
| TC <-> FC | 0.904 | 0.904 | 0.866 | 0.940 |
| SMA <-> AT | 0.915 | 0.915 | 0.877 | 0.951 |
| SP <-> SMO | 0.920 | 0.921 | 0.875 | 0.964 |
| SMO <-> SMA | 0.924 | 0.925 | 0.879 | 0.971 |
| SMA <-> MT | 0.937 | 0.937 | 0.902 | 0.969 |
| SP <-> SMA | 0.941 | 0.941 | 0.901 | 0.980 |
